# ID 126 - Podcast – O que é ATS e como ela impacta sua saúde?

**DOI:** 10.5327/2237-9622.2025.v34s1.126

**Published:** 2025-11-25

**Authors:** Clarissa Coelho Vieira Guimarães Coelho, Maykon Anderson Pires de Novais, Antônio Augusto de Freitas Peregrino, Lilian Reinaldi Ribeiro Pirozi, Maria Simone de Menezes Alencar, Márcia Rodrigues dos Santos

## Abstract

**Introdução:**

O podcast NATS-SPDM une os conceitos de inovação e saúde, ressaltando a importância da Avaliação de Tecnologias em Saúde (ATS) e dos Núcleos de Avaliação de Tecnologias em Saúde (Nats) no processo de análise e adoção de novas tecnologias pelos sistemas de saúde, evidenciando a conexão entre as instituições SPDM (Associação Paulista para o Desenvolvimento da Medicina) e Universidade Federal do estado do Rio de Janeiro (Unirio).

A apresentação será realizada por seis pesquisadores em temas de ATS, em uma dinâmica de conversa informal e envolvente, com a finalidade de disseminar o conteúdo para os alunos de graduação com interesse no tema e membros da sociedade objetivando a participação social. A abordagem visa utilizar uma linguagem acessível e próxima ao público jovem, ao mesmo tempo em que mantém a profundidade e a precisão das informações.

O episódio começa com uma breve explicação sobre o que é ATS destacando seu papel como um processo de avaliação dos benefícios, riscos e custos de novas tecnologias em saúde. Será discutido como a ATS impacta a escolha de medicamentos, equipamentos, procedimentos, entre outros, levando em consideração aspectos clínicos, econômicos e sociais.

A iniciativa busca conectar o ensino de ATS às práticas sociais criando um caminho menos formal para que novas tecnologias possam ser inseridas no sistema de saúde brasileiro de forma eficiente e consciente.

**Método:**

O processo de gravação e edição do podcast intitulado "O que é ATS e como ela impacta sua saúde?" envolveu a verificação de equipamentos e softwares essenciais para garantir a qualidade final do conteúdo.

O uso de um microfone de alta qualidade, acompanhado de um pop filter e um suporte adequado (braço ou pedestal), para a captação clara da voz, minimizando ruídos indesejados e distorções. Fones de ouvido de monitoramento foram importantes para a qualidade do áudio em tempo real durante a gravação e a edição.

A utilização de softwares de gravação, como Audacity, foi previamente configurada e testada, permitindo que o áudio fosse capturado com precisão. Além disso, a gravação num ambiente acusticamente controlado, livre de ecos e ruídos externos, para preservar a clareza do som. Elementos como trilhas sonoras e efeitos sonoros foram selecionados e ajustados para criar uma atmosfera envolvente e facilitar transições durante o episódio.

A plataforma de gravação digital selecionada foi o Zoom, oferecendo flexibilidade no processo. O software de edição escolhido é o editor de vídeo on-line do Clipchamp gratuito que permite ajustes finos na equalização e eliminação de imperfeições. Por fim, foi criado uma identidade visual sólida, com a criação de uma capa atrativa, desenvolvida em plataformas como Canva, que será utilizada para divulgação.

A etapa final consiste em garantir que a plataforma de publicação esteja configurada corretamente, utilizando serviços como Spotify para hospedar e distribuir o conteúdo, garantindo que o episódio seja acessível ao público-alvo. O método de desenvolvimento do podcast foi gravado com a participação de membros dos Nats envolvidos e utilizando um formato de conversa acessível e dinâmica. A partir de um script da gravação com duração de 30 minutos, a apresentação foi realizada por duas doutorandas da Universidade Federal do Estado do Rio de Janeiro e uma pós-doutoranda da Universidade Federal de São Paulo (Unifesp).

A estrutura foi dividida em três segmentos principais: introdução à ATS, conexão com a sustentabilidade, e iniciativas acadêmicas de democratização com o objetivo de educar os ouvintes sobre a ATS e mostrar como práticas sustentáveis podem ser integradas na avaliação de tecnologias de saúde. Os recursos de áudio e som utilizados durante as transições têm efeitos sonoros leves para criar uma atmosfera envolvente e agradável.

A abertura teve uma música instrumental suave e ambiental. Foi produzido uma identidade visual da logo do podcast, seguido de uma breve vinheta com o título do episódio. Será realizado a apresentação das professoras pelo locutor (): "Bem-vindos ao nosso podcast! Hoje, vamos falar sobre a democratização da Avaliação de Tecnologias em Saúde (ATS), com um enfoque sustentável. Para isso, temos a presença de três professoras que vão nos ajudar a entender melhor esse tema e discutir como ele pode impactar o futuro da saúde. Vamos começar!"

Foram elaboradas com cinco eixos: 1) início com pergunta provocativa; 2) explicação simples e acessível sobre ATS; 2) integração com os objetivos do SUS e da sustentabilidade; 3) papel dos estudantes; 4) o futuro da ATS; e 5) conclusão. Com essa iniciativa, queremos que os alunos de hoje sejam os profissionais de amanhã que entendam não só a importância da tecnologia, mas também como aplicá-la de forma que respeite o meio ambiente e os princípios do SUS. É um equilíbrio que precisamos construir desde já.

As perguntas elaboradas foram: Com tantas tecnologias sendo lançadas todos os dias, como podemos garantir que elas realmente melhorem o atendimento no SUS de maneira sustentável?

Pergunta 1: O que é ATS e por que democratizar? (2-10 min)

Som: Transição suave para o segmento.

"Para quem ainda não está familiarizado, a Avaliação de Tecnologias em Saúde (ATS) é o processo de analisar os benefícios, custos e impactos de novas tecnologias no campo da saúde. Mas por que é tão importante democratizar esse conhecimento entre os alunos de graduação?"

Professor(a) 1: "A democratização da ATS significa tornar acessível o entendimento sobre como as tecnologias são avaliadas e aprovadas. Isso permite que os alunos, que são futuros profissionais, compreendam desde cedo como decisões sobre saúde e tecnologia são feitas, e como essas escolhas afetam a sociedade."

Professor(a) 2:

"Além disso, ao envolver mais alunos nesse processo, criamos uma comunidade acadêmica mais consciente e preparada para contribuir com práticas inovadoras e sustentáveis. A inclusão desse tema nas graduações promove maior diversidade de pensamentos e soluções."

Pergunta 2: ATS e Sustentabilidade: Caminhos que se Cruzam (10-20 min)

Som: Sons leves

"Agora que entendemos a importância de democratizar a ATS, vamos explorar como isso se conecta com a sustentabilidade. Como a avaliação de tecnologias em saúde pode ser usada para promover práticas mais sustentáveis?"

Professor(a) 1:

"Exatamente. A ATS sustentável envolve pensar no ciclo de vida de uma tecnologia, desde sua fabricação até o descarte. Ao conscientizar os alunos sobre isso, estamos preparando uma geração que vai integrar saúde e sustentabilidade em suas práticas."

Professor(a) 2:

"Acho interessante que, ao discutir ATS nas aulas, também passamos a refletir mais sobre como podemos inovar com materiais recicláveis, energias renováveis e processos que minimizem os impactos negativos. É uma abordagem que nos prepara para o futuro."

Pergunta 3: Iniciativa de Democratização da ATS nas Universidades (20-28 min)

"Vocês poderiam explicar um pouco mais sobre a iniciativa de democratização da ATS nas universidades, e por que focar em práticas sustentáveis?"

Professor(a) 1:

"O objetivo dessa iniciativa é inserir a ATS no currículo de forma acessível, com workshops e aulas práticas onde os alunos podem simular processos de avaliação. Isso aproxima os estudantes das decisões que envolvem saúde pública e sustentabilidade."

Professor(a) 2:

"É uma proposta inovadora porque estamos integrando tecnologia, saúde e meio ambiente. Ao usar estudos de caso e dados reais, estimulamos o pensamento crítico nos alunos. A ideia é que eles saiam da universidade prontos para tomar decisões mais conscientes." Encerramento (28-30 min) Som: Música suave e otimista.

"E assim encerramos o nosso episódio de hoje! Agradecemos aos nossos convidados e a você, ouvinte, por nos acompanhar nessa discussão sobre democratização da ATS e sustentabilidade. Até o próximo episódio!"

